# MicroRNAs as potential biomarkers for monitoring of acquired sensorineural hearing loss

**DOI:** 10.1002/pdi3.51

**Published:** 2024-03-05

**Authors:** Yaqin Hu, Hongjiang Chen, Xiaoqing Zhou, Xiaoqin Luo, Zailan Tu, Jing Ke, Wei Yuan

**Affiliations:** ^1^ Chongqing Medical University Chongqing China; ^2^ Department of Otolaryngology Chongqing General Hospital Chongqing China

**Keywords:** biomarkers, circulating miRNAs, deafness, sensorineural hearing loss

## Abstract

Presbyacusis, sudden sensorineural hearing loss (SNHL), noise‐induced hearing loss, and drug‐induced deafness are the most common types of acquired SNHL. At present, the diagnosis of these hearing disorders is mainly based on the medical history and audiological examination. Due to the lack of diagnostic biomarkers, diagnosis of acquired sensorineural hearing loss is difficult. Previous studies have suggested that microRNAs (miRNAs) serve essential roles in pathophysiological processes, and they are stable, tissue enriched, and could be determined in a quantitative manner. Therefore, they could be potential noninvasive biomarkers for acquired SNHL. This review provided a comprehensive overview of the similarities and differences in altered circulating miRNAs (cimiRNAs) (plasma, serum, whole blood, and perilymph) in various disorders, and highlighted the regulatory functions of cimiRNAs on the development, monitoring and prognosis of the four most common types of acquired SNHL.

## 1 INTRODUCTION

Hearing loss is the one of the most common types of hearing impairments, which has become a major public health problem worldwide. World Health Organization (WHO) first reported that 42 million people (approximately 1% of the world's population) were estimated to have moderate to profound (or disabling) hearing loss in 1985,^1^ and by 2018, the estimate rose to 466 million. This estimate is projected to rise to 630 million by 2030 and to more than 900 million by 2050.^2^ The major types of hearing loss include conductive, sensorineural, and mixed hearing loss.^3^ Sensorineural hearing loss (SNHL) is the most common type of hearing impairment.^4^ SNHL is caused by damage or dysfunction of inner hair cells, auditory neurons synapses, or stria vascularis.^5^ Numerous factors are involved in the development of SNHL, such as noise, genetic mutations, aging, aminoglycoside antibiotics, and chemotherapy drugs.^3^ Because hearing loss can severely affect cognitive functions,^6^, ^7^ it is necessary to explore the underlying causes of hearing loss, and the treatments for this disorder could be developed as well. The diagnosis of SNHL is mainly based on audiological examination and the medical history. At present, novel biomarkers for acquired SNHL have been identified. Emerging evidence has indicated that microRNAs (miRNAs) serve essential roles on the progression of acquired SNHL, and they could be putative diagnostic and prognostic markers for this disease.^8^, ^9^

MiRNAs are a group of endogenous noncoding RNAs that contain 19–23 nucleotides. They are widely expressed in vertebrates, drosophila, nematodes, plants, and even viruses. MiRNAs regulate gene expression by means of base pairing with sites within the 3′‐untranslated region (3′‐UTR) of the target mRNA for target recognition.^10^ In fact, miRNAs are involved in various biological processes, including cell cycle, apoptosis, proliferation, and differentiation. Therefore, dysfunctions of miRNAs could result in pathophysiological effects,^11^ such as occurrence of tumors, cardiovascular diseases, and neurodegenerative disorders.^12^, ^13^, ^14^ At present, over 17,000 mature sequences of miRNAs have been identified.^15^ Moreover, previous studies have suggested the essential roles of miRNAs in the pathogenesis of hearing loss.^8^, ^16^

Most miRNAs are expressed inside the cells. However, numerous miRNAs have been detected in biological fluids of the human body such as blood, saliva, urine, and cerebrospinal fluid, which are called circulating miRNAs (cimiRNAs).^17^ cimiRNAs exhibit high stabilities in physiological fluids,^18^ and they are specifically expressed.^19^ Therefore, cimiRNAs could be more reliable biomarkers compared to other molecular markers. The abovementioned advantages make cimiRNA signature as an interesting biomarker candidate for the diagnosis, prognosis, and follow‐up of certain diseases, and they could be used as novel indicators for noninvasive screening.^20^ The aim of this review was to summarize the roles of cimiRNAs in the development, monitoring, and prognosis of acquired SNHL and identify miRNAs targets as novel biomarkers.

## 2 BIOGENESIS OF miRNAs

**FIGURE 1:**
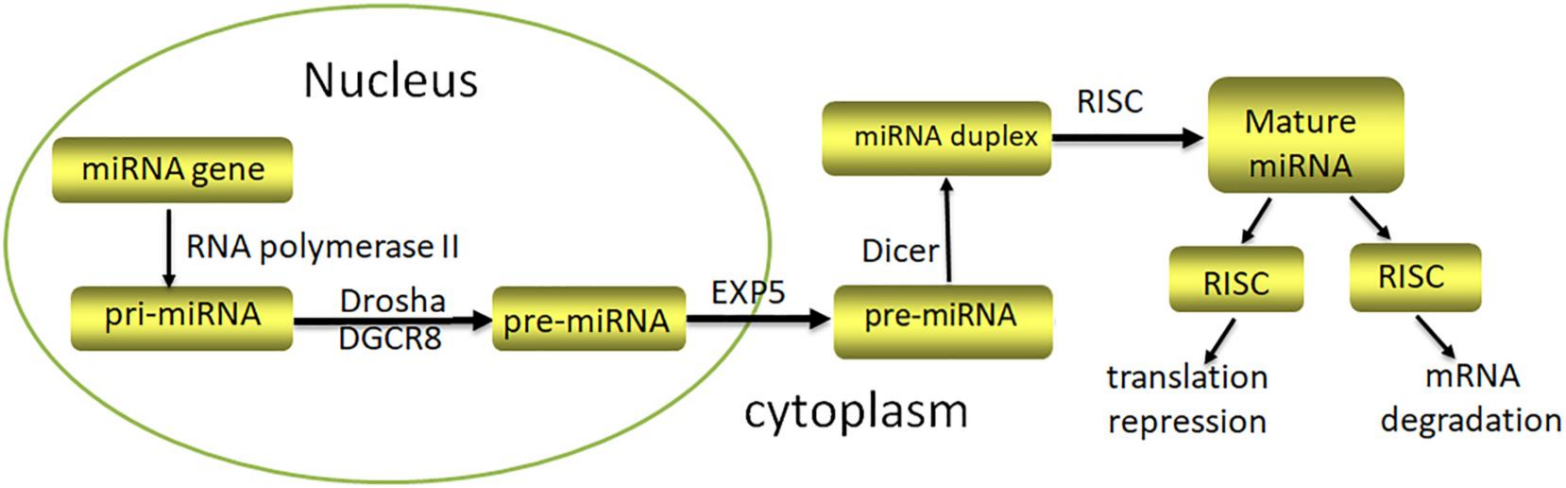
Biogenesis of miRNA. miRNA, microRNAs.

Biogenesis of miRNAs is a complex process involving multiple steps. This process occurs in the nucleus and cytoplasm, including transcription of primary transcript (pri‐miRNA), nuclear processing by Drosha, nucleocytoplasmic export, cytoplasmic processing by Dicer, and formation of RNA‐induced silencing complex (RISC).^21^ Firstly, the miRNA sequences are transcribed into pri‐miRNAs with a stem‐loop structure by RNA polymerase II. Secondly, pri‐miRNAs are processed into precursor miRNAs (pre‐miRNAs), which contain hairpin structure composed of RNase III family Drosha enzyme and double‐stranded RNA‐specific binding protein DGCR8. The length of pre‐miRNAs is 70–100 nucleotides. Thirdly, pre‐miRNAs are transported to cytoplasm by exportin‐5 and the following steps occur in the cytoplasm. Subsequently, pre‐miRNAs are cleaved into shorter double‐stranded miRNAs by the RNase III‐Dicer and transactivating response RNA‐binding protein complex. The length of miRNA duplex is 21–24 nucleotides. Within the two strands of miRNA duplex, only one strand forms miRNA‐induced silencing complex that contains members of Argonaute protein (Ago) family, while the other strand is degraded. RISC target complementary 3′‐untranslated regions (3′‐UTR) of mRNA transcripts, which can recognize target genes and subsequently mediate mRNA degradation or inhibit translation. In humans, precise pairing to the mRNA is not required, so each miRNA can regulate the expression of several target mRNAs, and vice versa; a single mRNA can be regulated by multiple miRNAs^22^, ^23^, ^24^ (Figure 1).

## 3 CimiRNAs AS POTENTIAL DIAGNOSTIC BIOMARKERS IN ACQUIRED SNHL

Recently, the expression of miRNAs has been detected not only within the cells but also in blood, saliva, urine, tears, and cerebrospinal fluid.^18^, ^25^ These miRNAs are collectively known as cimiRNAs. The emission process of miRNA usually occurs after cell death, for example, apoptosis, metastasis, or inflammation. It is now known that miRNAs are bound to protein, high‐density lipoprotein, and/or encapsulated in membrane‐bound vesicles protecting them from degradation,^23^, ^26^, ^27^ and they exhibit high stability even under severe conditions such as extreme pH, high temperature, prolonged storage time, and multiple freeze‐thaw cycles.^28^ CimiRNAs can be detected in plasma or other body fluids and they are differentially expressed during the development of certain diseases. In addition, collection of samples is minimally invasive or noninvasive, which allows repeated sampling. Due to the abovementioned characteristics, cimiRNAs could be used as potential biomarkers for numerous diseases such as heart failure,^29^ Alzheimer's disease,^30^ and major depressive disorders.^31^ Previous studies have also suggested that cimiRNAs could be potential biomarkers for acquired SNHL. This review focuses on four acquired SNHL, namely age‐related hearing loss (ARHL), sudden sensorineural hearing loss (SSNHL), noise‐induced hearing loss (NIHL), and drug‐induced deafness. We performed a PubMed search and explored all articles about SNHL. All included articles explored cimiRNA in acquired SNHL (Table 1). Exclusion and inclusion criteria for acquired SNHL are described and specified in each article.

**TABLE 1:** CimiRNAs as possible biomarkers for acquired sensorineural hearing loss.

MiRNAs	Model	Fluid	Disease	Method	Up/downregulation	Year
miR‐34	Human/mouse	Plasma	ARHL	qRT‐PCR	Upregulation	2016
miR‐183	Human	Plasma	SSNHL	qRT‐PCR/RNA‐seq	Upregulation	2020/2017
miR‐210	Human	Plasma	SSNHL	qRT‐PCR/RNA‐seq	Upregulation	2020/2017
miR‐23a	Human	Plasma	SSNHL	qRT‐PCR/RNA‐seq	Downregulation	2020/2017
miR‐18b	Human	Plasma	SSNHL	qRT‐PCR/RNA‐seq	Downregulation	2020/2017
miR‐185‐5p	Human	Plasma	NIHL	Microarray assay	Upregulation	2016
miR‐451a	Human	Plasma	NIHL	Microarray assay	Upregulation	2016
miR‐16‐5p	Human	Plasma	NIHL	Microarray assay	Upregulation	2016
miR‐24‐3p	Human	Plasma	NIHL	Microarray assay	Upregulation	2016
miR‐1229‐5p	Human	Serum	NIHL	Microarray assay/qRT‐PCR	Upregulation	2018
miR‐3162‐5p	Human	Serum	NIHL	Microarray assay	Upregulation	2108
miR‐4484	Human	Serum	NIHL	Microarray assay	Upregulation	2018
miR‐4652‐3p	Human	Serum	NIHL	Microarray assay	Downregulation	2018
miR‐205	Mouse	Serum	Drug‐induced deafness	qRT‐PCR	Upregulation	2018
miR‐183	Mouse	Serum	Drug‐induced deafness	qRT‐PCR	Upregulation	2018

Abbreviations: ARHL, age‐related hearing loss; NIHL, noise‐induced hearing loss; SSNHL, sudden sensorineural hearing loss.

## 4 CimiRNAs IN ARHL

Presbyacusis is type of age‐related decline in hearing acuity. It is one of the most common types of sensory hearing loss and is also known as ARHL. ARHL could be caused by genetic or environmental factors and cochlear aging. In 2005, the expression of miRNAs were first detected in the inner ear of zebrafish.^32^ Until now, accumulating evidence indicates that miRNAs serve essential roles on the maintenance of inner ear function.^33^ GeneChip miRNA microarray and real‐time PCR were used to examine the samples from C57 and CBA mice, and the results suggested that 182 miRNAs were differentially expressed.^34^ Furthermore, Sekine et al. suggested that the expression of 348 miRNAs was detected in human temporal bones; 277 miRNAs were downregulated and 71 miRNAS were upregulated during aging.^33^ These findings indicated that various genetic backgrounds might contribute to the dysregulation of miRNAs in ARHL.

During aging, downregulated miRNAs remarkably outnumber upregulated ones; downregulation of miRNAs such as the miR‐183 and miR‐181 family is known to be the key regulator of cell proliferation and differentiation.^34^ Moreover, upregulated miRNAs including the members of miR‐29 and miR‐34 family are involved in the pro‐apoptotic pathways such as p53, p27, and Bcl2 signaling.^35^ Additionally, the miR‐183 family which consists of miR‐96, miR‐182, and miR‐183 are regulatory factors during the development of cochlea.^36^, ^37^ Furthermore, a previous study has revealed that mutations in miR‐96 suppressed the morphological development of hair cell stereocilia through affecting cell length, synaptic active zone, and innervation pattern.^38^ Lewis et al. have suggested that miR‐182 knockout mice exhibited progressive hearing loss; mice carrying null alleles of miR‐183/96 were completely deaf, whose hair cell stereocilia were abnormal and numbers of inner hair cell synapses were reduced at 4 weeks old.^39^ The miR‐183 family has been widely studied due to their important roles on hair cell differentiation. However, Shew et al. have revealed that the expression of miR‐183 was not detected in human inner ear perilymph.^40^ Until now, the involvement of the members of miR‐183 family in blood or other fluids during the progression of presbycusis is still not confirmed.

The miR‐181 family containing miR‐181a‐5p, miR‐181b‐5p, miR‐181c‐5p, and miR‐181d‐5p is evolutionarily well conserved across all vertebrate species.^41^ The members of miR‐181 family serve essential roles in numerous physiological and pathological processes such as the neurotrophin signaling, axon guidance, immune response, and mitochondrial‐related pathways.^42^, ^43^, ^44^ A previous study has suggested that the members of miR‐181 family are downregulated in C57BL/6J and CBA/J mice during aging.^34^ In addition, miR‐181a‐5p has been detected in human inner ear perilymph.^40^ These studies have suggested that the expression of miR‐181 family is associated with the development of ARHL. Although it has been reported that the miR‐181 family are expressed in the blood and cerebrospinal fluid of patients with age‐related diseases^45^; the detailed functions of circulating miR‐181 during the progression of presbycusis still remain unclear.

Until now, the level of circulating miR‐34a is the only miRNA indicator of ARHL that could be potential biomarker for early detection of this disease.^3^ MiR‐34 is a highly conserved miRNA expressed in many organisms.^46^ Previous studies have indicated that the expression of miR‐34 increases with age.^34^, ^47^ Yang et al. have reported that loss‐of‐function mutation in miR‐34 remarkably delays age‐related physiological decline, extends lifespan, and enhances resistance to heat and oxidative stress in *Caenorhabditis elegans*.^48^ SIRT1, Bcl‐2, and E2F3 are targets of miR‐34a. Xiong et al. examined the molecules involved in the miR‐34a/Sirtuin 1 (SIRT1)/p53 signaling in cochlear hair cells during aging; the results revealed that miR‐34a expression, p53 acetylation, and cell apoptosis were increased, while SIRT1 expression was decreased in the cochlea of C57BL/6 mice during aging.^49^ Furthermore, their findings suggested that the miR‐34a/SIRT1 signaling protected cochlear hair cells from oxidative stress and delays ARHL through coordinated regulation of mitophagy and mitochondrial biogenesis.^50^ Huang et al. revealed the upregulation of miR‐34a and downregulation of Bcl‐2 in the cochlea of C57BL/6 mice during aging, suggesting that suppression of Bcl‐2 by miR‐34a could promote age‐induced apoptosis in hair cells.^51^ Pang et al. revealed that miR‐34a levels were significantly higher in patients with presbycusis compared with controls; however, the expression of SIRT1, Bcl‐2, and E2F3 in plasma was not affected.^52^ Although the causes of upregulated miR‐34a in ARHL remain largely unknown, the results in this study revealed that miR‐34a might be a novel biomarker for early detection of presbycusis.^52^

## 5 CimiRNAs IN SSNHL

SSNHL is defined as a SNHL of 30 dB or greater over three contiguous audiometric frequencies occurring within 3 days, and the etiology is unclear.^53^ It is a type of acquired SNHL. The average age of first occurrence of SSNHL is typically mid‐40s to mid‐50s. Prevalence rates do not differ by gender, season, or geographic region.^54^ The pathogenesis of SSNHL remains largely unknown.

RNA sequencing on the plasma results of Li et al. have indicated that 24 differentially expressed microRNAs (DEMs) between the SSNHL group and control group; they found that hsa‐miR‐34a/15a/23a/210/18b/548n/143 serve essential roles during the development of SSNHL.^55^ The data of GO annotation analysis have suggested that the target genes of these abovementioned miRNAs are mainly involved in the biological processes such as salivary gland maturation, neurotransmission, and dendritic development; the results of KEGG pathway enrichment analysis have revealed that they are functionally enriched in coagulation cascades, arachidonic acid metabolism, the MAPK signaling, and linoleic acid metabolism.^55^ Nunez et al. used TaqMan low‐density array on serum miRNAs in 36 SSNHL patients and 12 controls. Their research found that eight miRNAs were different.^56^ They are Hsa‐miR‐5p/186‐5p/195‐5p/140‐3p/128‐3p/132‐3p/375‐3p/30a‐3p and most of them were abundantly identified in the nervous system. We can see that there are difference between the results of the two reports, which may be caused by the difference of race or body fluid types. However, the two studies indicate that cimiRNAs is expressed differentially between SSNHL patients and controls. Ha et al. carried out a further research, they have revealed in their research that the levels of miR‐183, miR‐210, miR‐23a, and miR‐18b can be used to determine the degree of SNHL and predict the therapeutic outcome.^57^

Moreover, miR‐183 served key roles during the development of hair cells.^40^ It is abundantly expressed in the hair cells of inner ear in adults.^58^ MiR‐210 is involved in numerous biological processes, such as DNA repair, angiogenesis, cell cycle, and immune response.^59^ It is upregulated in cells under hypoxic conditions. Riccardi et al. have indicated that the upregulation of miR‐210 promote the differentiation of epithelial cells into sensory hair cells.^60^ The upregulation of circulating miR‐183 and miR‐210 could be caused by the damage of hair cells. The detailed roles of miR‐23a and miR‐18b in hearing still remain largely unknown. A previous study has revealed that circulating miR‐23a and miR‐18b are downregulated in patients with SNHL,^56^ but the underlying mechanisms are unclear. Human miR‐23a is located at chromosome 19, and it is the first member of the miR‐23a∼27a∼24‐2 miRNA cluster.^61^ Shew et al. have revealed that miR‐23a is expressed in human inner ear perilymph.^40^ Moreover, MDM2 is the target gene of miR‐18b,^62^ and the disruption of the MDM2/p53 interaction affects the early‐embryonic otic progenitor cells and their descendants, the auditory supporting cells, and hair cells.^63^

## 6 CimiRNAs IN NIHL

NIHL is considered as a type of SNHL caused by gene–environment interactions.^64^ Occupational noise‐induced hearing loss (ONIHL) is a common type of occupational diseases. The pathogenesis of NIHL is still unclear. Although ONIHL is caused by long‐term and continuous exposure to excessive noise, individual sensitivity to noise exposure could be different due to genetic variations.^65^ Recently, research have been carried out to identify the susceptibility genes of NIHL.^66^, ^67^ These genes have been divided into four groups: genes involved in the oxidative stress pathways, internal potassium recycling, monogenic deafness genes, and heat‐shock protein‐associated genes.^68^

MiRNAs serve essential roles during the progression of NIHL via regulating their target genes.^69^, ^70^ Previous studies have suggested that miRNAs could be aberrantly expressed under adverse environmental and pathological conditions, and they could be potential biomarkers to indicate the responses to noise exposure or the occurrence of NIHL.^71^, ^72^ Microarray analysis by Lu et al. have identified 73 differentially expressed miRNAs in response to noise exposure between ONIHL patients and normal controls.^71^ Furthermore, they have revealed that miR‐185‐5p and miR‐451a are upregulated in plasma samples from NIHL patients. The expression of miR‐185 is increased under oxidative stress, subsequently promoting DNA damage‐associated apoptosis.^73^ In addition, the upregulation of miR‐185 could reduce the expression of androgen receptor protein, consequently inducing cell cycle arrest at G0/G1 phase in vitro.^74^

MiR‐451a is a member of the miR‐451 family.^75^ MiR‐451 is expressed in human cochlea^33^ and inner ear perilymph.^40^ A previous study revealed the key roles of miR‐451 in response to oxidative stress.^75^ MiR‐451 protect cardiomyocyte against oxidative stress‐induced damage through upregulating Rac‐1 and the CUGBP2 (CUG triplet repeat‐binding protein 2)‐COX‐2 signaling.^76^, ^77^ Wang et al. have revealed that miR‐451 is upregulated in an HEI‐OC1 cell model treated with tert‐butyl hydroperoxide.^78^ Moreover, the upregulation of circulating miR‐451 could be associated with oxidative stress that is the main inducer of NIHL.

In addition, Li et al. conducted miRNAs microarray analysis on serum of ONIHL subjects and controls. They revealed that there were three upregulations (hsa‐miR‐3162‐5p, hsa‐miR‐4484 and hsa‐miR‐1229‐5p) and one downregulation (hsa‐miR‐4652‐3p) among these miRNA in serum of ONIHL group. They further verified this by real time quantitative PCR and found that serum miRNA‐1229‐5P was significantly upregulated in ONIHL subjects compared to controls.^72^ MAPK1 is identified as a novel target of miR‐1229‐5p using bioinformatics analysis and luciferase dual reporter assay. MAPK1 is a key regulator of the survival of hair cells in NIHL, and it prevents the hearing loss caused by noise exposure in mice.^79^

## 7 CimiRNAs IN DRUG‐INDUCED DEAFNESS

Until now, more than 200 drugs with ototoxicity have been reported.^80^ Ototoxic drugs can cause symptoms such as tinnitus, vertigo, hyperacusis, and hearing loss by affecting the functions of cochlea or vestibule. Aminoglycoside antibiotics are the most commonly used ototoxic drugs that destroy sensory hair cells by causing mitochondrial damage, consequently leading to permanent deafness.^81^ In clinical practice, the therapeutic outcomes of hearing loss treated with aminoglycoside antibiotics vary a lot. Some patients treated with aminoglycoside antibiotics do not exhibit the symptoms of ototoxicity, while some may experience hearing loss even at the usual therapeutic dose.

Additionally, the time of disease onset is unpredictable. Some patients experience hearing loss after a single standard dose, whereas some may not exhibit the symptoms of hearing loss until the treatment is completed. Sun et al. have revealed that cimiRNA‐205 and miRNA‐183 can be detected in the ototoxicity‐induced mouse model using qRT‐PCR, and circulating miR‐205 reaches the maximum level on day 3 and remains at a constant level from day 7 to day 14.^82^ These findings suggest that circulating miR‐205 in the blood could be used to predict the degree of hearing loss when ototoxic drugs are used. MiRNA‐205 is abundantly expressed in different types of epithelial tissues such as breast, prostate, skin, eyes, and thymus.^83^ Moreover, ROS is closely associated with the signaling pathways involved in aminoglycoside antibiotics‐involved diseases. A previous study has indicated that miR‐205 could protect cells from oxidative stress by suppressing EGLN2 and subsequently downregulating intracellular ROS.^84^ Sun et al. have suggested that the upregulation of circulating miR‐205 is associated with aminoglycoside antibiotics‐induced damage to the inner ear, and circulating miR‐205 in the cochlea migrates through the blood vessels to other organs after ototoxic damage.^82^

## 8 CONCLUSIONS AND PERSPECTIVES

MiRNAs serve essential roles on biological processes such as cell differentiation, proliferation, autophagy and apoptosis. Moreover, the expression of certain miRNAs are cell‐specific^85^ and exhibit high extracellular stability.^86^ The reports of using cimiRNAs as biomarkers are increasing in recent years. However, CimiRNA as a biomarker for acquired SNHL has not been widely reported. At present, a few studies have reported the expression differences of cimiRNA in acquired SNHL. In ARHL, the levels of cimiR‐34a in plasma were increased and it could be used as potential novel biomarker for early detection of this disease in future; however, the underlying mechanisms remained largely unknown. In sudden SNHL, the levels of circulating miR‐183, miR‐210, miR‐23a, and miR‐18b could be used to identify the disease state and predict the therapeutic efficacy; the sensitivity and specificity of combined evaluation were 80.95% and 87.50%, respectively.^56^ In NIHL, the expression of circulating miR‐185‐5P and miR‐451a was upregulated in the plasma, suggesting that they could be putative diagnostic markers for NIHL.^71^ In drug‐induced deafness, the levels of circulating miR‐205 in the blood could indicate the degree of hearing loss and inner ear damage,^81^ which might be attributed to the involvement of miR‐205 in oxidative stress. These studies suggested that cimiRNAs may help in revealing the complex pathophysiological mechanisms of acquired SNHL.

Although the reports of cimiRNAs are increased in recent years, cimiRNAs are still in their infancy as a reliable biomarker for acquired SNHL. This review shows that many factors, such as bodily fluid, age, gender, methods, and techniques, affect the expression level of cimiRNA. Therefore, larger sample sizes and more studies are needed to validate these cimiRNAs. In addition, standard processes and methods for detecting miRNA are also important. With the development of the field of cimiRNAs, there could be novel biomarkers for acquired SNHL in the future; some of them might be used for early diagnosis and evaluating prognosis of therapies of this diseases in future clinical practice.

## AUTHOR CONTRIBUTIONS

**Yaqin Hu**: writing—original draft; preparation. **Hongjiang Chen**: Data curation; Conceptualization; writing—review and editing. **Xiaoqing Zhou**: writing—review and editing; formal analysis. **Xiaoqin Luo**: editing. **Zailan Tu**: editing. **Jing Ke**: writing—review and editing. **Wei Yuan**: Conceptualization; supervision; writing—review and editing.

## CONFLICT OF INTEREST STATEMENT

The authors declare no conflicts of interest.

## ETHICS STATEMENT

Not applicable.
